# Medicines for eye health

**Published:** 2023-05-22

**Authors:** Fatima Kyari, Elmien Wolvaardt

**Affiliations:** Associate Professor: International Centre for Eye Health, London School of Hygiene & Tropical Medicine, UK.; Consultant Ophthalmologist: College of Health Sciences, University of Abuja, Nigeria.; Editor-in-Chief: Community Eye Health Journal, ICEH, LSHTM, London, UK.

**Teamwork is vital if we want to support patients to access and use their eye medication.**

**Figure:**
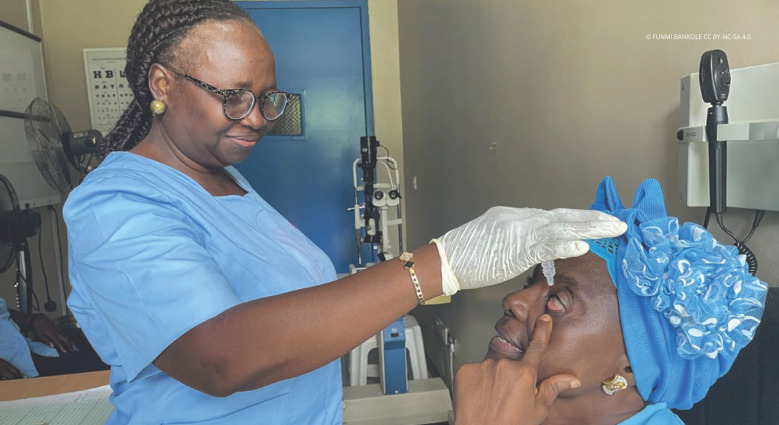
Medicines play a vital role in eye care. nigeria

Medicines have a vital role in eye care, not only when patients are in the hospital or eye clinic, but also when they have to continue caring for their eyes at home.

For patients to adhere with the prescribed use of eye medicines, two conditions must be met:

1. The medication prescribed must be locally available, at a cost the patient can afford (also in the long term if they have a chronic eye condition). To address this, we have included articles on advocacy, local production of eye drops, and the management of eye medicines in a hospital or clinic setting.
2. Patients must also understand how and why to use their eye medication. We therefore have an article on empowering patients and a one-page instruction sheet which you can copy and share directly with patients.

Each member of the eye care team has a role in meeting these two conditions, and good communication is essential.

- **Pharmacists** can check prescriptions for safety and accuracy, give feedback to clinicians, and support patient education and adherence.
- **Clinicians and prescribers** can ask pharmacists which medicines are locally available and affordable.
- **Nurses and allied health personnel** can support patients by checking they have the correct prescription and/or medication before leaving the hospital, and by teaching them how to use their medication correctly.
- **Managers and policy makers** can support advocacy efforts or consider producing eye drops locally.

However, as we hope to show in this issue, these eye care team members will be most effective in their role if they can recognise that the patient is the most important member of the eye care team. If the patient's needs, abilities, circumstances and preferences are not taken into account at every step, adherence will remain a challenge and even the best medication will not be effective.

